# Hormesis, cell death and aging

**DOI:** 10.18632/aging.100380

**Published:** 2011-09-08

**Authors:** Isabelle Martins, Lorenzo Galluzzi, Guido Kroemer

**Affiliations:** ^1^ INSERM, U848, 94805 Villejuif, France; ^2^ Institut Gustave Roussy, 94805 Villejuif, France; ^3^ Université Paris Sud XI, 94270 Le Kremlin-Bicêtre, France; ^4^ Metabolomics Platform, Institut Gustave Roussy, 94805 Villejuif, France; ^5^ Centre de Recherche des Cordeliers, 75006 Paris, France; ^6^ Pôle de Biologie, Hôpital Européen Georges Pompidou, AP-HP, 75015 Paris, France; ^7^ Université Paris Descartes, Faculté de Médecine, 75006 Paris, France

## Abstract

Frequently, low doses of toxins and other stressors not only are harmless but also activate an adaptive stress response that raise the resistance of the organism against high doses of the same agent. This phenomenon, which is known as “hormesis”, is best represented by ischemic preconditioning, the situation in which short ischemic episodes protect the brain and the heart against prolonged shortage of oxygen and nutrients. Many molecules that cause cell death also elicit autophagy, a cytoprotective mechanism relying on the digestion of potentially harmful intracellular structures, notably mitochondria. When high doses of these agents are employed, cells undergo mitochondrial outer membrane permeabilization and die. In contrast, low doses of such cytotoxic agents can activate hormesis in several paradigms, and this may explain the lifespan-prolonging potential of autophagy inducers including resveratrol and caloric restriction.

## INTRODUCTION

Hormesis (a neologism coined from the ancient Greek term *hormáein*, which literally means “to set in motion, impel, urge on”) describes a favorable biological response to harmless doses of toxins and other stressors. Hormesis-stimulating compounds initiate an adaptive stress response that renders cells/organisms resistant against high (and normally harmful) doses of the same agent. On the theoretical level, hormesis may constitute (one of) the mechanisms that allows stressed cells to avoid senescence and death, and hence might have some impact on the (patho)physiology of aging. Thus, measures that reportedly prolong the healthy lifespan of multiple species, such as caloric restriction and the administration of resveratrol [1-6], may do so by inducing a hormetic response [7,8]. In this article, we will examine the molecular circuitries that link cellular stress and death, and how these pathways can get uncoupled during hormetic responses.

### Redundant pathways leading to apoptotic cell death

Apoptosis is frequently viewed as a caspase-dependent cell death pathway in which a series of specific cysteine proteases are activated in a cascade of proteolytic maturation steps [9,10]. In response to cell death-inducing signals, so-called initiator caspases (*i.e.*, caspase-8 and -9) [11,12] get engaged and activate so-called effector caspases (*i.e.*, caspase-3, -6 and -7) [13], which in turn degrade multiple proteins causing the arrest of vital cellular functions as well as the initiation of lethal catabolic reactions [14-16]. Two upstream events account for the activation of initiator caspases. In the extrinsic pathway, caspase-8 is recruited to and gets activated within the death-inducing signaling complex (DISC), a multiprotein complex that forms at the cytoplasmic tails of a specific class of cell surface receptors, the death receptors, upon their occupancy by their respective ligands [12,17-19]. In the intrinsic pathway, caspase-9 is activated at the so-called apoptosome, a supramolecular entity that involves dATP, the cytoplasmic protein APAF1 and the mitochondrial intermembrane space factor cytochrome *c*, and that only forms when the outer mitochondrial membrane, which usually separates APAF1 (outside) and cytochrome *c* (inside) is permeabilized [20-24].

Nonetheless, caspase inhibition rarely prevents cell death completely (although it sometimes attenuates the morphological manifestations of apoptosis) [10,25-27], and multiple caspase-independent pathways may come into action [9,28,29]. For example, permeabilized mitochondria can allow for the release of apoptosis-inducing factor (AIF) and cofilin, both of which have been shown to operate as caspase-independent death effectors [30-33]. This places the control of cell death at the level of the mitochondrion, and more precisely at the level of mitochondrial membranes, whose permeabilization is controlled by multiple upstream effectors and processes, including distinct classes of stress-activated kinases [24,34-43], the tumor suppressor protein p53 [44-46], epigenetic perturbations [47-51], (de)acetylases [52,53], perturbations of the cell cycle [54,55], and nuclear damage [56-60].

In accord with the key role of mitochondria in the regulation of many (if not all) apoptotic pathways [61-65], mitochondrial functions and integrity are controlled by a variety of distinct mechanisms. Proteins from the Bcl-2 family are considered as central modulators of mitochondrial apoptosis [66-68], but other proteins that are not directly related to BCL-2 can induce or suppress mitochondrial outer membrane permeabilization (MOMP) as well. Thus, beyond BCL2 and its close relatives BCL2L1 (best known as Bcl-X_L_) and MCL1, PRELI [69], the uncoupling protein 2 (UCP2) [70] and the X-linked inhibitor of apoptosis protein (XIAP) [22] can prevent MOMP. Pro-apoptotic proteins like the multidomain proteins BAX and BAK [71], as well as multiple BH3-only proteins stimulate MOMP [72-74]. In addition, pro-oxidants [75-77], membrane-destabilizing lipids (such as ceramide) [52] and free Ca^2+^ ions (modulated by other divalent cations such as Mg^2+^ and Zn^2+^, and possibly by the monovalent cation Li^+^) [21,23,78-80] can stimulate MOMP.

### Autophagy as a cytoprotective mechanism

Macroautophagy (to which we refer to as “autophagy”) is a lysosomal degradation pathway in which portions of the cytoplasm (organelles or cytosol) are enwrapped in double-membraned vesicles (called autophagosomes) that fuse with lysosomes and get degraded by lysosomal hydrolases [81-84]. Importantly, autophagy and apoptosis exhibit a consistent degree of crosstalk, at multiple levels [85-87].

Autophagy can lead to the removal of damaged, potentially dangerous mitochondria, thereby increasing the threshold for cell death induction by MOMP-inducing agents or other stressors. Thus, both mitochondrion-specific autophagy (mitophagy) and general autophagy can reduce the propensity of cells to undergo apoptosis [1,76,88-90].

Caspase-dependent apoptosis is associated with the degradation of Beclin 1 by caspases. As Beclin 1 is essential for the initial steps of autophagy, caspase activation most often result into the inhibition of the autophagic pathway [16,91,92]. This reflects a general pattern according to which pro-apoptotic signals result in the inhibition of pro-survival systems.

**Figure 1:**
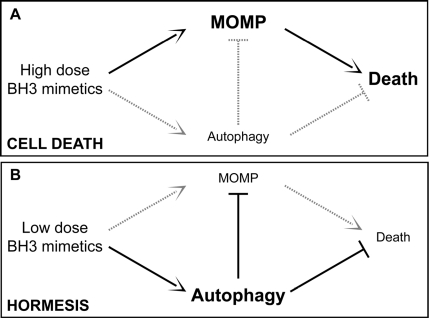
Autophagy and hormesis In baseline conditions, autophagy contributes to the maintenance of cellular homeostasis by removing potentially dangerous mitochondria (or other damaged organelles) and by ensuring the disposal of protein aggregates. This might have anti-aging effects and prolong healthy lifespan by elevating the threshold of damage required for the induction of cellular dysfunctions or death. In this scenario, while high doses of agents that stimulate both autophagy and cell death (*e.g.*, BH3 mimetics) would be toxic (**A**), low doses of the same agents might provoke a hormetic response and hence favor the adaptation of cells to stress (**B**). MOMP, mitochondrial outer membrane permeabilization.

Some molecular mechanisms that sense cellular stress can induce both autophagy and apoptosis. This applies for instance to BH3 proteins (as well as pharmacological BH3 mimetics), which can liberate Beclin 1 from inhibitory interactions with BCL-2-like proteins, thereby favoring autophagy, and also stimulate MOMP by activating BAX or BAK [93-101]. It is thought that the relative abundance of different Bcl-2 family members, as well as their subcellular localization and activation state may determine whether BH3 proteins/mimetics induce autophagy or apoptosis [66,67]. Moreover, endoplasmic reticulum stress can either result in autophagy or in apoptosis, depending on a yet-to-elucidated interplay among threshold effects [88,102,103]. One possible scenario suggests that mild stress would induce an autophagic response that elevates the threshold for apoptosis induction. This would represent a typical case of hormesis (**Figure 1**).

### Autophagy as an anti-aging mechanism

Pharmacological or genetic manipulations designed to prolong lifespan induce autophagy in multiple model organisms, including yeast, nematodes and flies, and the inhibition of autophagy often (always?) prevents longevity extension in such settings. This applies to lifespan extension induced by caloric restriction, genetic or pharmacological activation of Sirtuin 1, inhibition of the mammalian target of rapamycin (mTOR) with rapamycin, and administration of spermidine, a histone acetylase inhibitor [3,4,81,104-107]. Among these stimuli, there is circumstantial evidence that Sirtuin 1 (whose activation occurs during and is necessary for starvation- and resveratrol-induced autophagy) acts in a hormetic fashion. One of the best-known systems of hormesis is ischemic preconditioning (IPC), whereby short episodes of ischemia protect the brain against a later, more severe reduction in oxygen and nutrient supply. In this system, the administration of resveratrol can mimic IPC, and both resveratrol and IPC induce similar changes in the acetylproteome of the brain [53].

It is currently a mystery through which mechanisms autophagy may increase lifespan [108,109]. One obvious possibility resides in the cytoprotective, apoptosis-inhibitory action of autophagy (see above), although other possibilities must be considered as well. For example, there is ample evidence that the inhibition of mTOR (which stimulates autophagy, yet may have other metabolic effects as well) antagonizes senescence [105,110-115]. Thus, it can be speculated, yet remains to be proven, that autophagy would avoid cellular senescence induced by DNA-damaging agents [116-119], as well as the attrition of stem cells that accompanies advanced aging [120-123]. Simultaneously, there is strong evidence indicating that autophagy functions as an onco-suppressive mechanism that avoids the genetic instability that accelerates multi-step oncogenesis [3,114,124-130].

In addition, autophagy enhances the turnover of aggregate-prone cellular proteins, thus reducing the abundance of potent neurotoxic factors including, but not limited to, huntingtin aggregates [131]. Whether the induction of autophagy may also affect the accumulation of potentially toxic extracellular proteins (such as amyloid β and others) remains to be clarified [32,132-134].

At this stage, it is not clear which (if any) among these putative mechanisms plays a preponderant role in the longevity-increasing potential of autophagy. Future work will have to clarify this issue, which may have a major impact on how we design strategies for prolonging healthy lifespan.

## Abbreviations

AIF: apoptosis-inducing factor
DISC: death-inducing signaling complex
IPC: ischemic preconditioning
MOMP: mitochondrial outer membrane permeabilization
UCP2: uncoupling protein 2
XIAP: X-linked inhibitor of apoptosis protein
